# High-resolution population structure and runs of homozygosity reveal the genetic architecture of complex traits in the Lipizzan horse

**DOI:** 10.1186/s12864-019-5564-x

**Published:** 2019-03-05

**Authors:** Gertrud Grilz-Seger, Thomas Druml, Markus Neuditschko, Max Dobretsberger, Michaela Horna, Gottfried Brem

**Affiliations:** 1 Institute of Animal Breeding and Genetics, Department for Biomedical Sciences, University of Veterinary Medicine Vienna, Veterinärplatz 1, A-1210 Vienna, Austria; 2 Agroscope, Swiss National Stud Farm, Les Longs Prés, CH-1580 Avenches, Switzerland; 3 Department of Animal Husbandry, Slovak University of Agriculture in Nitra, Nitra-Chrenová, Slovak Republic

**Keywords:** Lipizzan horse, Runs of homozygosity (ROH), ROH islands, NetView, Melanoma, HOXB cluster, selection signature

## Abstract

**Background:**

The sample ascertainment bias due to complex population structures remains a major challenge in genome-wide investigations of complex traits. In this study we derived the high-resolution population structure and levels of autozygosity of 377 Lipizzan horses originating from five different European stud farms utilizing the SNP genotype information of the high density 700 k Affymetrix Axiom™ Equine genotyping array. Scanning the genome for overlapping runs of homozygosity (ROH) shared by more than 50% of horses, we identified homozygous regions (ROH islands) in order to investigate the gene content of those candidate regions by gene ontology and enrichment analyses.

**Results:**

The high-resolution population network approach revealed well-defined substructures according to the origin of the horses (Austria, Slovakia, Croatia and Hungary). The highest mean genome coverage of ROH (S_ROH_) was identified in the Austrian (S_ROH_ = 342.9), followed by Croatian (S_ROH_ = 214.7), Slovakian (S_ROH_ = 205.1) and Hungarian (S_ROH_ = 171.5) subpopulations. ROH island analysis revealed five common islands on ECA11 and ECA14, hereby confirming a closer genetic relationship between the Hungarian and Croatian as well as between the Austrian and Slovakian samples. Private islands were detected for the Hungarian and the Austrian Lipizzan subpopulations. All subpopulations shared a homozygous region on ECA11, nearly identical in position and length containing among other genes the homeobox-B cluster, which was also significantly (*p* < 0.001) highlighted by enrichment analysis. Gene ontology terms were mostly related to biological processes involved in embryonic morphogenesis and anterior/posterior specification. Around the *STX17* gene (causative for greying), we identified a ROH island harbouring the genes *NR4A3*, *STX17*, *ERP44* and *INVS*. Within further islands on ECA14, ECA16 and ECA20 we detected the genes *SPRY4*, *NDFIP1*, *IMPDH2*, *HSP90AB1*, whereas *SPRY4* and *HSP90AB1* are involved in melanoma metastasis and survival rate of melanoma patients in humans.

**Conclusions:**

We demonstrated that the assessment of high-resolution population structures within one single breed supports the downstream genetic analyses (e.g. the identification of ROH islands). By means of ROH island analyses, we identified the genes *SPRY4*, *NDFIP1*, *IMPDH2*, *HSP90AB1*, which might play an important role for further studies on equine melanoma. Furthermore, our results highlighted the impact of the homeobox-A and B cluster involved in morphogenesis of Lipizzan horses.

**Electronic supplementary material:**

The online version of this article (10.1186/s12864-019-5564-x) contains supplementary material, which is available to authorized users.

## Background

The Lipizzan horse breed is globally one of the best-documented horse population, as pedigree records can be traced back to the known founder animals born in the early eighteenth century. The founder population, described in detail by Zechner et al. [1], Druml and Sölkner [2] and Druml et al. [3], comprises 456 animals, whereas the major part of horses originated from the former imperial stud farm of Lipica founded in the year 1580. Since the First World War the stud book of Lipizzan horses is closed and presently conservation breeding strategies are applied by eleven state stud farms located in nine European countries. National Lipizzan breeding herds have limited population sizes; therefore, maintenance of genetic diversity has been in the focus of state stud farms over decennia.

Numerous genetic approaches and methods have been applied to characterise the gene pool, the genetic diversity and the population structure of the Lipizzan breed. Comprehensive pedigree data (max. 31 generations, generation equivalent 19.1) were used to estimate inbreeding coefficients and effective population sizes by Zechner et al. [1], Sölkner and Druml [2] and Druml et al. [3]. Furthermore, microsatellite markers were employed to describe diversity measures, genetic distances and population structure by Achmann et al. [4]. Kavar et al. [5, 6] investigated mtDNA maternal diversity; Kasarda et al. [7] estimated genetic relatedness between Old Kladruber, Slovenian and Slovakian Lipizzans, whilst Wallner et al. [8] highlighted the patrilinear structure conducting haplotype-based analyses of the Y-chromosome. Most of these scientific publications arose from a multilateral research project, which is described in the review by Dovc et al. [9]. Based upon this scientific project further research was conducted focusing on the inheritance of melanoma, vitiligo and greying [10–13].

In this study we used the SNP genotype information of the Affymetrix Axiom™ Equine genotyping array [14] to analyse the high-resolution population structure of Lipizzan horses originating from five different European stud farms, which differ in breeding history and breeding objectives. To ascertain the high-resolution population structure of the gene pool, we applied a recently described three-step procedure as presented in Druml et al. [15], which includes individual levels of admixture and genomic inbreeding (ROH) in the final population network visualization. After the assessment of the high-resolution population structure, we identified overlapping homozygous regions (ROH islands) within the entire population and respective subpopulations, and conducted a gene ontology and enrichment analysis of annotated genes located within ROH islands. We demonstrate that the combination of different approaches elaborate insights in population structure and underlying differences in levels of autozygosity and distribution of ROH islands.

## Results

### Genetic diversity and admixture analysis

**Fig. 1:**
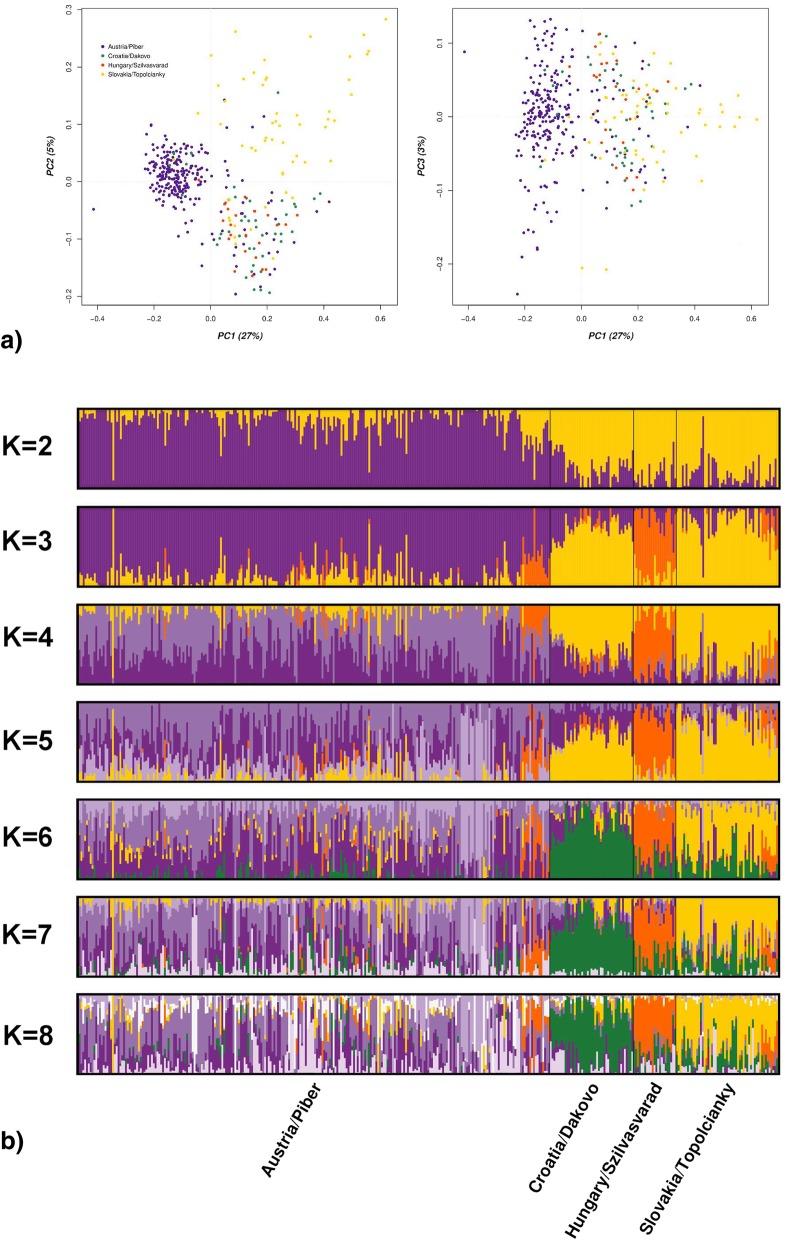
Visualization of the dataset on the first 3 Principal Components (PCs) together explaining 35% of the variation. **a** On the left plot of PC1 and PC2; on the right plot of PC1 and PC3. **b** Graphical representation of individual cluster membership coefficients for Admixture runs increasing *K* = 2–8 for the Austrian, Croatian, Hungarian and Slovakian sample of Lipizzan horses

Principal Component analysis (PCA) as presented in Fig. 1a) revealed that the first three Principal Components (PCs) accounted for 35% of the total genetic variance. Based upon the visualization of the first three PCs the Austrian population is characterized by lower pairwise genetic distances and a distinct clustering. The other three subpopulations (Croatia, Hungary and Slovakia) appear to be highly interrelated simultaneously expressing a high level of genetic diversity, whilst a subset of the Austrian samples shares similarities with Croatian, Slovakian and Hungarian horses. F_ST_ analysis recapitulated these findings, as nearest relationships were documented for Austrian, Croatian and Slovakian samples (pairwise F_ST_ from 0.025 to 0.045), whereas the Hungarian subpopulation exhibited higher genetic differentiation (pairwise F_ST_ from 0.068 to 0.092) (Additional file 1).

The first level (K = 2) of model-based clustering using the programme Admixture separated the Austrian population from the other Lipizzan samples (Fig. 1b). At the second level of clustering K = 3 the Hungarian sample were allocated in a distinct cluster. Further increasing K to 4 and 5 the Austrian subpopulation was further sub-structured, whilst at K = 6 the Slovakian sample formed a distinct cluster. At the additional levels of K = 7 and K = 8 the identified subpopulations were subsequently sub-structured. The visualization of the cross-validation (CV) error for each K, increasing K from 2 to 10, did not result in an optimal number of clusters (Additional file 2).

### High-resolution network visualisation

**Fig. 2:**
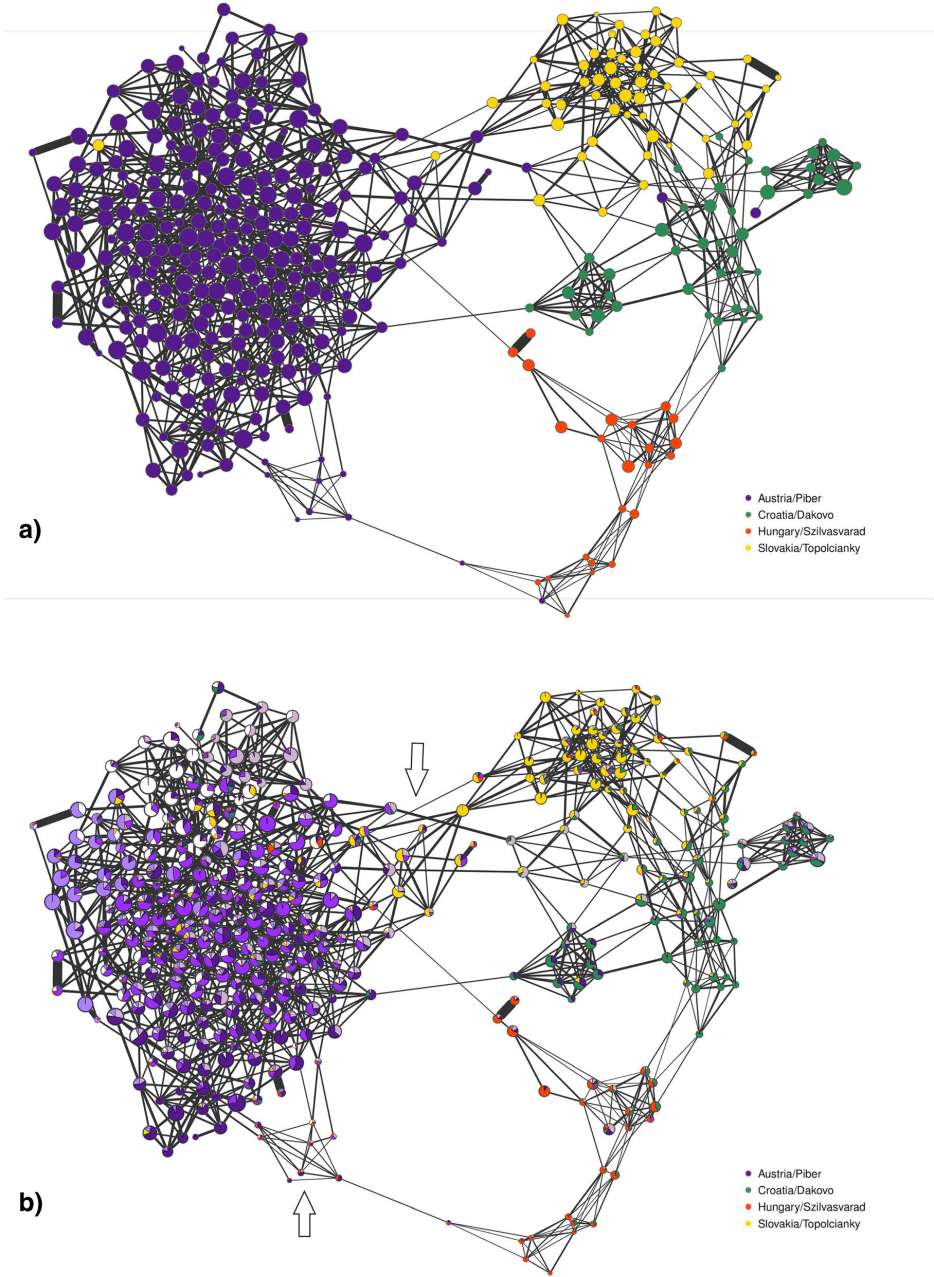
High-resolution network visualisation of Lipizzan horses from the stud farms Piber (Austria), Topol’čianky (Slovakia), Đakovo and Lipik (Croatia) and Szilvasvárad (Hungary). **a** On the top network the horses (nodes) are colored by stud farm. On the lower network (**b**) nodes are containing individual cluster membership coefficients from Admixture analysis. Each node represents an individual and the size of node is in relation to S_ROH_, link lengths between nodes illustrate the genetic distance between individuals, thickness of links are proportional to genetic relationship. Two arrows pinpoint sub-clusters in the Austrian sample

Using a high-resolution network graph we combined individual levels of S_ROH_ (see the results below) and individual levels of admixture (K = 8). The network analysis improved the assessment of the population structure, compared to PCA and Admixture, by clearly separating the horses according to their origin, simultaneously highlighting four population outliers (Fig. 2). The horses of the stud farm Piber characteristically were connected by short and thick links, representing higher co-ancestry and lower genetic distance. The network highlighted two sub-clusters with higher levels of admixture, which were linked to Slovakian and Hungarian Lipizzans (marked with arrows in Fig. 2). According to pedigree information these cross-link animals were foreign bred mares, which were integrated into the Piber breeding population and thus are characterized by a lower co-ancestry level (longer and thinner links) and smaller S_ROH_ values (smaller node sizes).

The Croatian sample was separated from the Austrian sample, connected directly by one cross-link animal. The network analysis identified three sub-clusters within the Croatian sample. According to pedigree information, the cluster on the right involved horses from the stud farm Lipik, whilst the left cluster represented the core population from the stud farm Đakovo. The accumulation in the middle of the Croatian sample included horses from Đakovo, which showed high levels of admixture with horses from Lipik, as well as with Slovakian and Hungarian Lipizzans, respectively. The Hungarian sample was characterised by the highest genetic distances between the samples and by the lowest S_ROH_ values, indicating genealogical separation between Hungarian and Austrian Lipizzans. The Slovakian sample showed genetic relationships in both directions – to the Austrian and to the Croatian samples. These relationships were illustrated by manifold cross-link animals, which had small S_ROH_ values and higher degree of admixture connecting both clusters.

### Runs of homozygosity

Including the entire sample of Lipizzan horses in the ROH analysis the mean number of ROH (N_ROH_) comprised 202.07 segments at a mean genome length covered by ROH (S_ROH_) of 297.01 Mb (+ − 119.85), resulting in a mean ROH length (L_ROH_) of 1.42 Mb (Table 1).

**Table 1:** Mean values, standard deviation (SD), minimum and maximum values for the variables S_ROH_ (in Mb), N_ROH_, L_ROH_ (in Mb) and F_ROH_ for the entire Lipizzan sample, the Austrian population sample of the stud farm Piber, the population of the Slovakian stud farm of Topol’čianky, the Croatian and the Hungarian sample

Total sample	n	mean	SD	Min.	Max.
S_ROH_	377	297.01	119.85	1.91	550.21
N_ROH_		202.07	51.96	3.00	333.00
L_ROH_		1.42	0.43	0.56	2.45
F_ROH_		0.13	0.05	0.00	0.25
Piber/Austria					
S_ROH_	254	342.88	105.43	3.35	550.21
N_ROH_		211.85	46.00	6.00	333.00
L_ROH_		1.59	0.41	0.56	2.45
F_ROH_		0.15	0.05	0.00	0.25
Topol’čianky/Slovakia					
S_ROH_	55	205.05	76.75	30.38	359.72
N_ROH_		178.58	50.81	48.00	301.00
L_ROH_		1.06	0.17	0.63	1.35
F_ROH_		0.09	0.03	0.01	0.16
Lipik, Đakovo/Croatia					
S_ROH_	45	214.66	85.99	81.31	401.22
N_ROH_		186.87	47.38	96.00	320.00
L_ROH_		1.11	0.22	0.85	1.62
F_ROH_		0.10	0.04	0.04	0.18
Szilvasvárad/Hungary					
S_ROH_	23	171.53	112.58	1.91	402.80
N_ROH_		158.39	83.30	3.00	131.00
L_ROH_		0.98	0.22	0.64	1.44
F_ROH_		0.07	0.05	0.00	0.18

ROH parameters varied between samples. Highest estimates of S_ROH_ and N_ROH_ were identified for horses originating from the stud farm of Piber/Austria (S_ROH_ = 342.88 Mb; N_ROH_ = 211.85), followed by the Croatian horses (S_ROH_ = 214.66 Mb; N_ROH_ = 186.87) and Slovakian horses (S_ROH_ = 205.06 Mb; N_ROH_ = 187.58). Lowest S_ROH_ and N_ROH_ were found in the Hungarian sample (S_ROH_ = 171.53 Mb; N_ROH_ = 158.39) (Table 1). Five Austrian Lipizzan horses reached the highest S_ROH_ with more than 500 Mb, whereas the smallest S_ROH_ were detected in three Hungarian Lipizzan horses (1.91 Mb to 20 Mb).

Inbreeding as defined by F_ROH_ reached a mean value of 13.2% (+ − 5.3%) within the entire sample, ranging from 15.3% (+ − 4.7%) in the Piber population to 7.6% (+ − 5.0%) in the Hungarian sample (Table 1). To differentiate old and recent inbreeding we calculated F_ROH_ based upon different ROH length classes (Table 2). The values for F_ROH < 2Mb_ were highest in the Austrian (6.7%) and lowest in the Hungarian sample (5.6%). To quantify recent inbreeding we calculated F_ROH_ considering ROHs longer than 10 MB. Recent inbreeding was absent in the Slovakian sample and tended to zero in the Hungarian sample, whereas the highest value of 1.1% was observed for the Austrian sample.

**Table 2:** Distribution of ROHs and F_ROH_ (in percent) in different length classes (0.5–1 Mb, 1–2 Mb, 2–4 Mb, 4–6 Mb, 6–8 Mb, 8–10 Mb, > 10 Mb) respectively cumulative length classes (< 1 Mb, < 2 Mb, < 4 Mb, < 6 Mb, < 8 Mb, < 10 Mb, total F_ROH_) for the Austrian, Croatian, Hungarian, Slovakian samples and the entire data set (All)

Distribution ROH in length classes	Austria	Croatia	Hungary	Slovakia	All
0.5–1 Mb	22.7	37.7	41.0	40.0	28.1
1–2 Mb	21.3	31.1	32.1	32.3	24.8
2–4 Mb	23.9	22.2	19.4	21.4	23.1
4–6 Mb	12.9	5.7	5.2	4.8	10.4
6–8 Mb	7.6	2.2	1.2	0.9	5.6
8–10 Mb	4.2	0.4	0.4	0.5	3.0
> 10 Mb	7.4	0.6	0.6	0.0	5.1
F_ROH_ per length class					
F_ROH 0.5 -1Mb_	3.5	3.6	3.1	3.7	3.5
F_ROH 1-2Mb_	3.3	3	2.5	3	3.1
F_ROH 2-4Mb_	3.7	2.2	1.5	2	3.1
F_ROH 4-6Mb_	2	0.6	0.4	0.4	1.5
F_ROH 6-8Mb_	1.2	0.2	0.1	0.1	0.8
F_ROH 8-10Mb_	0.6	0	0	0	0.4
F_ROH > 10Mb_	1.1	0.1	0	0	0.8
F_ROH_ per length class (cum.)					
F_ROH < 1Mb_	3.5	3.6	3.1	3.7	3.5
F_ROH < 2Mb_	6.7	6.5	5.6	6.6	6.6
F_ROH < 4Mb_	10.4	8.7	7.1	8.6	9.7
F_ROH < 6Mb_	12.4	9.3	7.5	9	11.2
F_ROH < 8Mb_	13.5	9.5	7.6	9.1	12
F_ROH < 10Mb_	14.2	9.5	7.6	9.1	12.5
F_ROH total_	15.3	9.6	7.6	9.1	13.2

ROH patterns as revealed by the plot N_ROH_ versus S_ROH_ indicate for the majority of the Piber population a strong right shift towards a higher S_ROH_ and an increased variance of S_ROH_ (Additional file 3). The samples of Slovakia and Croatia were distributed along the diagonal, whereas in the left corner of the plot a cluster of 11 horses with the lowest ROH parameters can be observed, indicating out-breeding. These horses were also clearly identified as cross-link animals between stud farms within the high-resolution population network (Fig. 2).

Concerning the distribution of ROH segments of different length categories, the Austrian Lipizzan population is characterized by the lowest proportion of ROH smaller than 2 Mb (21.3%), which varied in the other samples between 31.1 and 32.3% (Table 2). At the same time the Austrian horses had the highest proportion of ROHs longer than 6 Mb (19.2%). In the other samples this ROH category was present at an amount from 1.4 to 3.2%. In the Austrian sample 7.4% of ROHs were longer than 10 Mb, whereas in the other samples this category was underrepresented at a percentage of < 0.6%.

### ROH islands

**Fig. 3:**
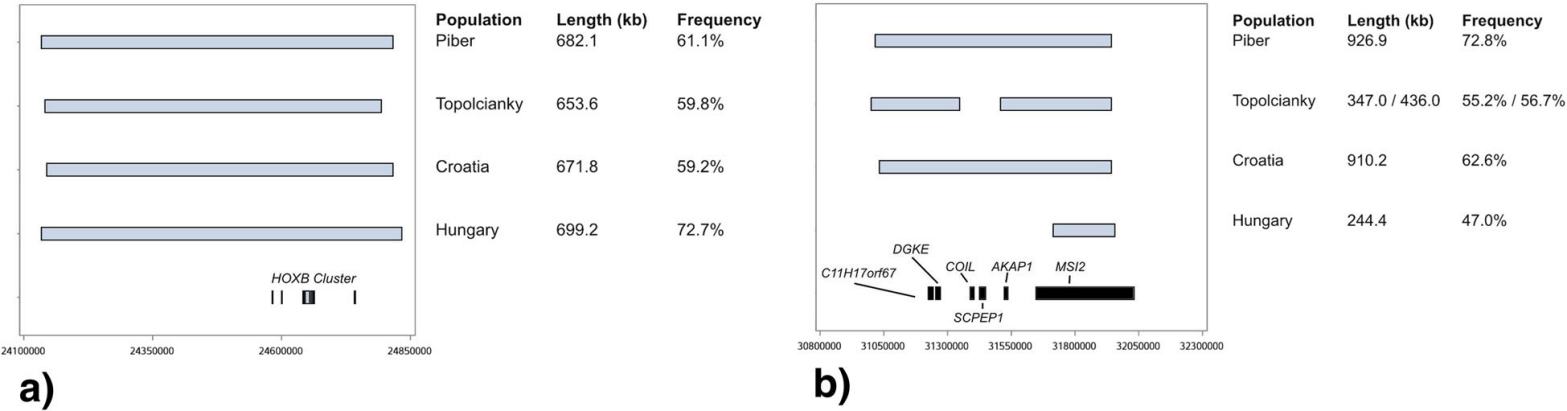
**a** Plot of the ROH islands containing the HOXB cluster at ECA11:24.13–24.81 Mb for each single sample. In Figure **b**) the island on ECA11:31.01–31.94 Mb including the genes *C11H17orf67, DGKE, COIL, SCPEP1, AKAP1, MSI2* is shown

Within the entire dataset, we identified three ROH islands on ECA11 and two islands on ECA14, which were shared by more than 50% of the horses (Table 3). The overlapping homozygous region on ECA11:24.13–24.81 Mb was shared by 61.3% of animals and harboured the genes *COPZ2, CBX1, SNX11, HOXB1, HOXB2, HOXB3, HOXB5, HOXB6, HOXB7, HOXB8, HOXB13, TTLL6.* Figure 3a illustrates this island and the position of the homeobox-B cluster (*HOXB)* for each sample separately. These overlapping homozygous regions were nearly identical in position and length in all samples and the frequency varied between 59.2% (Slovakia) and 72.7% (Hungary).

**Table 3:** ROH islands, shared by more than 50% (ROH freq.) of the entire sample of Lipizzan horses and the annotated genes in corresponding regions

Chr.	Begin	End	Length (kb)	ROH freq.	Known genes
11	24,134.125	24,816.254	682.1	0.613	*COPZ2, CBX1, SNX11, HOXB1, HOXB2, HOXB3, HOXB5, HOXB6, HOXB7, HOXB8, HOXB13, TTLL6*
11	30,677.562	30,685.590	8.0	0.501	*–*
11	31,015.821	31,942.748	926.9	0.666	*C11H17orf67, DGKE, COIL, SCPEP1, AKAP1, MSI2*
14	34,988.691	34,999.535	10.8	0.501	–
14	34,651.534	34,935.341	283.8	0.514	*SPRY4, NDFIP1*

The second ROH island on ECA11 (position 31,015.821 - 31,942.748) was up to 927 kb long in Austrian and Croatian samples and was shared by 72.8 and 62.6% of the horses, respectively (Fig. 3b). The Slovakian Lipizzans had two overlapping homozygous segments in this region. Within the Hungarian sample, 47.0% of horses shared a much smaller island, which was directly located in the Musashi RNA binding protein 2 (*MSI2)*.

**Fig. 4:**
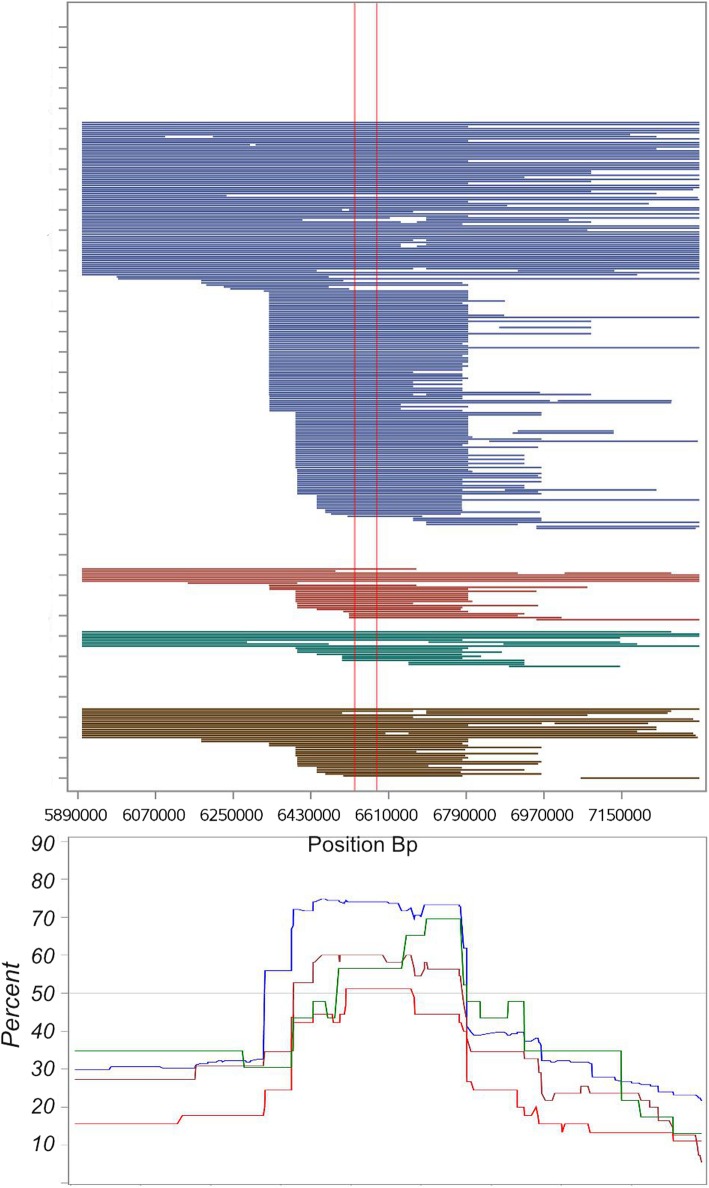
ROH island around the *STX17* gene (begin and end illustrated by red vertical reference lines) at ECA25:6.394.110–6.794.044. Subpopulations are presented in different colours: Austria = blue, Slovakia = brown, Croatia = red, Hungary = green

We identified a ROH island on ECA25 around the *STX17* (syntaxin-17) gene responsible for grey coat colour, shared by 46.2% of horses within the entire sample, containing the genes *NR4A3*, STX17, *ERP44* and *INVS*. This ROH island was embedded in the centre of a 1.38 Mb long homozygous region at position ECA25:5.69–7.07 Mb present in 36.6% of Lipizzan horses. The frequency was below our expectations. From a previous study, it is known that the *STX17* genotype frequencies for homozygous *G/G* horses reached up to 67.3% in Lipizzans [13]. Scanning this region for ROHs shorter than 500 kb, we applied a smaller boundary (80 kb window length, min. 20 SNPs), and detected a 399.9kb long ROH island at position ECA25:6394.110-6794.044 centred round the *STX17* gene that was shared by 66.1% of the entire sample (Fig. 4). Across the subpopulations the frequencies for this ROH island was highest in the Austrian sample (71.2%) and lowest in the Croatian sample (51.1%).

Considering the Austrian sample we found 16 ROH islands, which were located on ECA3 (2 islands), ECA5, ECA7 (2 islands), ECA8, ECA11 (3 islands), ECA14, ECA16 (4 islands), ECA18 and ECA20 (Table 4). The ROH islands ranged in length between 20.2 kb and 953.3 kb, whereas the longest fragment on ECA11 at 31.01–31.94 Mb was shared by the highest number of individuals (72,8%), followed by the ROH island on ECA11 at position 24.13–24.81 Mb, which was observed in 61,1% of animals. Within the Austrian sample eight ROH islands on ECA3, ECA5, ECA8, ECA11, ECA14, ECA16, ECA18 and ECA20 were private.

**Table 4:** ROH islands, shared by more than 50% (ROH freq.) of the Lipizzan horses from the Austrian stud farm Piber and the annotated genes in corresponding regions

Chr.	Begin	End	Length (kb)	ROH freq.	Known genes
3	118,669.793	118,769.903	100.1	0.508	*UVSSA, MAEA*
3	118,809.979	118,893.880	83.9	0.506	*CTBP1*
5	48,702.703	49,211.103	508.4	0.530	*CHD1L, FMO5, PRKAB2*
7	50,434.281	50,606.416	172.1	0.507	–
7	50,636.963	50,733.100	96.1	0.508	–
8	93,409.518	93,605.837	196.3	0.538	*CTDP1*
11	24,134.125	24,816.254	682.1	0.611	*COPZ2, CBX1,SNX11, HOXB1, HOXB2, HOXB3, HOXB5, HOXB6, HOXB7, HOXB8, HOXB13, TTLL6*
11	30,607.721	30,687.266	79.6	0.526	*–*
11	31,015.821	31,942.748	926.9	0.728	*C11H17orf67, DGKE, COIL, SCPEP1, AKAP1, MSI2*
14	41,779.891	42,365.786	585.9	0.546	*FSTL4*
16	37,870.208	38,823.469	953.3	0.542	*C16H3orf84, KLHDC8B, CCDC71, LAMB2, USP19, QARS, QRICH1, IMPDH2, NDUFAF3, DALRD3, ARIH2, WDR6, P4HTM, IP6K2, NCKIPSD, CELSR3, TMEM89, UQCRC1, SLC26A6, UCN2, PFKFB4, SHISA5, TREX1, ATRIP, CCDC51, PLXNB1, NME6, ECATH-3, ECATH-2*
16	39,675.098	39,695.300	20.2	0.507	*–*
16	39,890.148	39,920.855	30.7	0.507	*CCDC12*
16	40,077.806	40,159.633	81.8	0.514	*TMIE, ALS2CL*
18	635.232	1235.687	600.5	0.528	*ARHGEF4, PLEKHB2*
20	43,065.487	43,166.223	100.8	0.507	*CAPN11, SLC29A1, HSP90AB1, SLC35B2, NFKBIE, TCTE1*

Within the Slovakian sample we detected six ROH islands on ECA11 (3), ECA14, ECA16 and ECA22, whereas the islands on ECA11 and ECA16 overlapped with corresponding islands of the Austrian Lipizzans (Table 5). The ROH islands on ECA22:47.90–48.48 Mb and ECA14:34.65–35.08 Mb were also identified in the Croatian sample.

**Table 5:** ROH islands, shared by more than 50% (ROH freq.) of the Lipizzan horses from the Slovakian stud farm Topol’čianky and the annotated genes in corresponding regions

Chr.	Begin	End	Length (kb)	ROH freq.	Known genes
11	24,140.999	24,793.573	652.6	0.598	*COPZ2, CBX1,SNX11, HOXB1, HOXB2, HOXB3, HOXB5, HOXB6, HOXB7, HOXB8, HOXB13, TTLL6*
11	31,000.312	31,347.338	347.0	0.552	*C11H17orf67, DGKE*
11	31,507.758	31,942.748	435.0	0.567	*AKAP1, MSI2*
14	34,651.534	35,086.649	435.1	0.609	*SPRY4, NDFIP1, GNPDA1, RNF14*
16	39,675.098	39,775.712	100.6	0.507	*SETD2*
22	47,905.031	48,488.839	583.8	0.576	*CDH4, TAF4, LSM14B, PSMA7, SS18L1, MTG2, HRH3, OSBPL2, ADRM1, LAMA5, CABLES2, RBBP8NL, GATA5*

Within the Croatian sample six ROH islands on ECA4, ECA7, ECA11 (2 islands), ECA14, ECA22, which varied in length between 129.1 kb and 1.14 Mb, were identified. The longest overlapping homozygous region on ECA14:34.05–35.18 Mb was shared by 72.6% of horses and contained nine annotated genes (Table 6).

**Table 6:** ROH islands, shared by more than 50% (ROH freq.) of the Lipizzan horses from the Croatian stud farms Lipik and Đakovo and the annotated genes in corresponding regions

Chr.	Begin	End	Length (kb)	ROH freq.	Known genes
4	58,110.547	58,620.749	510.2	0.529	*HOXA1, HOXA2, HOXA3, HOXA5, HOXA6, HOXA7, HOXA9, HOXA10, HOXA11, EVX1, HIBADH*
7	52,014.500	52,143.583	129.1	0.532	*–*
11	24,144.431	24,816.254	671.8	0.592	*COPZ2, CBX1,SNX11, HOXB1, HOXB2, HOXB3, HOXB5, HOXB6, HOXB7, HOXB8, HOXB13, TTLL6*
11	31,032.580	31,942.748	910.2	0.626	*C11H17orf67, DGKE, COIL, SCPEP1, AKAP1, MSI2*
14	34,051.957	35,189.508	1137.5	0.726	*ARHGAP26, FGF1, SPRY4, NDFIP1, GNPDA1, RNF14, PCDH12, PCDH1, DELE1*
22	47,901.830	48,624.259	722.4	0.549	*TAF4, CDH4, LSM14B, PSMA7, SS18L1, MTG2, HRH3, OSBPL2, ADRM1, LAMA5, CABLES2, RBBP8NL, GATA5, SLCO4A1, NTSR1*

The island on ECA4 at position 58.11–58.62 Mb was also present in the Hungarian Lipizzans and contained among other annotated genes the homeobox-A cluster (*HOXA*). Furthermore, an island on ECA22 at position 47.90–48.62 Mb was exclusively detected within the Croatian and Slovakian sample.

**Fig. 5:**
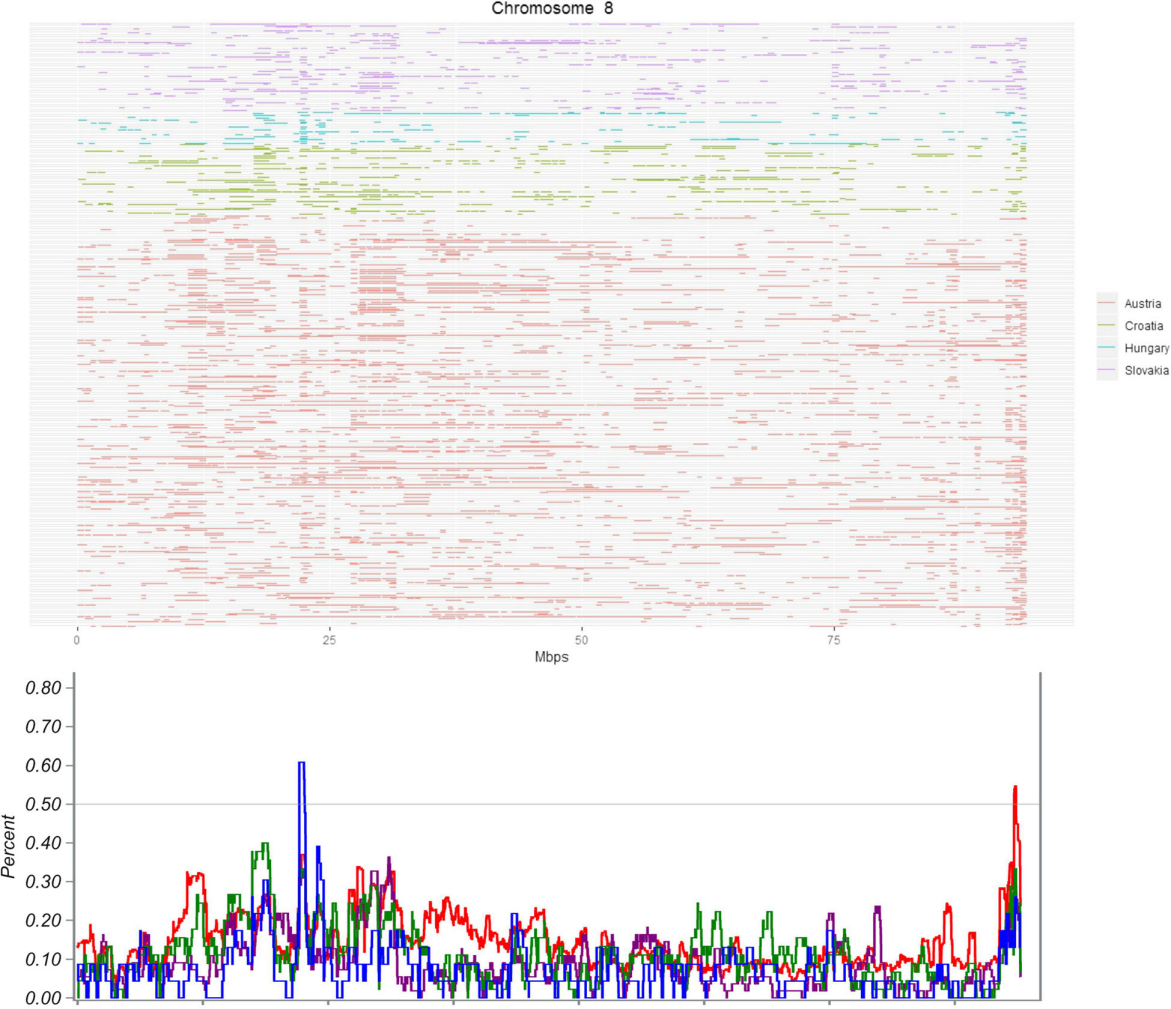
ROH distribution on ECA8 for the entire Lipizzan sample containing one private ROH island for the Hungarian sample (blue line) and one ROH island for the Austrian sample (red line) in the line plot below

Within the Hungarian Lipizzans six ROH islands on ECA4, ECA8, ECA11, ECA22, ECA23, ECA30 were identified, varying in length between 497.4 kb and 699.2 kb (Table 7). Four ROH islands on ECA8, ECA22, ECA23 and ECA30 were specific for the Hungarian sample. On ECA8 also a private island of the Austrian sample was documented (Fig. 5).

**Table 7:** ROH islands, shared by more than 50% (ROH freq.) of the Lipizzan horses from the Hungarian stud farms Szilvasvárad and the annotated genes in corresponding regions

Chr.	Begin	End	Length (kb)	ROH freq.	Known genes
4	58,107.774	58,605.189	497.4	0.522	*HOXA1, HOXA2, HOXA3, HOXA5, HOXA6, HOXA7, HOXA9, HOXA10, HOXA11, EVX1*
8	22,121.802	22,651.589	529.8	0.602	*TMEM120B, RHOF, SETD1B, HPD, PSMD9, WDR66, BCL7A, MLXIP, IL31, LRRC43, B3GNT4, DIABLO, CLIP1*
11	24,134.125	24,833.366	699.2	0.727	*COPZ2, CBX1,SNX11, HOXB1, HOXB2, HOXB3, HOXB5, HOXB6, HOXB7, HOXB8, HOXB13, TTLL6, CALCOCO2*
22	45,518.067	46,096.341	578.3	0.522	*ZNF831, EDN3, PHACTR3*
23	27,658.254	28,189.490	531.2	0.522	*UHRF2, GLDC, KDM4C*
30	4781.530	5465.214	683.7	0.522	*KIF26B, SMYD3*

### Gene ontology and enrichment analysis

Gene ontology and enrichment analysis highlighted within all Lipizzan samples the homeobox-B cluster, where the highest significance levels (*p* < 0.001) were reached for the terms GO:0048704~embryonic skeletal system morphogenesis and GO:0009952~anterior/posterior pattern specification (Additional file 4). Further the term GO:0043565~sequence-specific DNA binding related to molecular functions reached a significance level of p < 0.001 for the entire Lipizzan sample. Three out of 20 genes located in the identified ROH islands were listed in OMIM (Online Mendelian Inheritance in Man) database *(DGKE* - 615,008~Hemolytic uremic syndrome; *HOXB1*–614744~Facial paresis; *SPRY4*–615266~Hypogonadotropic hypogonadism), but none of the identified genes were listed in OMIA (Online Mendelian Inheritance in Animals) database for horses.

In the Austrian Lipizzan population (Additional file 5) GO analysis confirmed the aforementioned terms in biological processes (GO:0048704 and GO:0009952 containing the *HOXB* cluster), and highlighted additionally the five terms GO:0021570~rhombomere 4 development, GO:0021612~facial nerve structural, GO:0006183~GTP biosynthetic process, GO:0006950~response to stress and GO:0071353~cellular response to interleukin-4, related to biological processes.

The Slovakian Lipizzans (Additional file 6) where characterized by three specific terms related to biological processes: GO:0048864~stem cell development, GO:0001525~angiogenesis, GO:0006368~transcription elongation from RNA polymerase II promoter.

GO analysis revealed 12 identical terms, related to biological processes for the Hungarian and Croatian samples and additionally two sample specific terms (GO:0006355~regulation of transcription, DNA-templated; GO:0001578~microtubule bundle formation) for the Hungarian and three terms for the Croatian sample (GO:0007156~homophilic cell adhesion via plasma membrane adhesion molecules; GO:0050890~cognition; GO:0001759~organ induction) (Additional files 7 and 8). All these 12 terms related to biological processes are based upon the *HOXB*- and *HOXA*-clusters. The highest significance levels (*p* < 0.001) were found in concordance with the other samples for GO:0009952~anterior/posterior pattern specification and GO:0048704~embryonic skeletal system morphogenesis. Summarizing the results from gene ontology analysis of these two samples a high number of terms were highlighted for different aspects of embryonic morphogenesis (f.e. GO:0009953~dorsal/ventral pattern formation, GO:0009954~proximal/distal pattern formation, GO:0008584~male gonad development, GO:0060065~uterus development, GO:0021615~glossopharyngeal nerve morphogenesis).

### Single SNP analysis

Two islands in the entire sample and six islands in the Austrian Lipizzans were remarkable short (8.0–101.1 kb length) and matched in size with single genes. One short island on ECA3:118.67–118.77 Mb (length 101.1kb) directly overlapped with the genes *UVSSA* (UV-stimulated scaffold protein A) and *MAEA* (macrophage erythroblast attacher) and was shared by 50.8% of the Austrian horses. 76 kb upstream a second ROH island (ECA3:118.80–118.89 Mb, length 83.9kb) was centred on the *CTBP1* gene (C-terminal binding protein 1), that was shared by 50.2% of Austrian Lipizzans. To examine levels of homozygosity for SNPs located within the three named genes, we extracted the following SNPs from the entire sample: *UVSSA*: AX-104358355 Pos.:118,712.729, AX-104277358 Pos.:118,716.414, AX-103191894 Pos.:118,724.527, AX-104669126 Pos.:118,727.858; *MAEA:* AX-104046583 Pos.:118,750.308, AX-104041685 Pos.:118,752.167; *CTBP1:* AX-103592395 Pos.:118,874.036. The four SNPs within *UVSSA* revealed homozygosity for 78.2% of individuals. For the two SNPs located in *MAEA* 90.5% of animals were homozygous in the Austrian population and 78.5% of the horses were homozygous for these SNPs within the entire sample. At the SNP located in *CTBP1* all horses of the entire sample were homozygous.

## Discussion

The high-resolution population network (Admixture and Netview analysis) of the Lipizzan horse breed revealed that the single stud farms represent differentiated subpopulations, whereas the highest genetic distances were observed between the Austrian and the Hungarian/Croatian samples. The Slovakian Lipizzans clustered between the Austrian and the Croatian Lipizzans. These findings are in concordance with Achmann et al. [4], who derived a comparable structure for 561 Lipizzans from seven European countries. In the present study the Austrian and Slovakian horses were characterized by a lower genetic distances and higher pairwise relationship coefficients compared to the Croatian and Hungarian animals. This can be explained by the limited census of breeding animals in the Slovakian stud farm Topol’čianky, and the limited level of introgression of horses into the Austrian breeding herd. Due to the obligatory performance test of stallions in the Spanish riding school, in the Austrian stud farm Piber only foreign mares can be used to increase genetic diversity, whereas in Hungary, Slovakia and Croatia an exchange of breeding stallions is commonly applied. At the same time the Croatian and Hungarian breeding stock has been bigger than the Slovakian one.

Generally genetic distances between horses, illustrated in the high-resolution network visualization, was relatively equally distributed within the breeding herds of Piber and Topol’čianky and no outstanding contribution of single animals (key-contributor) were detected, revealing the efforts of a conservation breeding program with narrow sex ratios and moderate selection intensity. Between Piber, Szilvasvárad and Topol’čianky cross-link animals were found, which showed markedly smaller mean genome-wide coverage of ROH and a higher degree of admixture. A comparable “outbreeding-effect” was detected in a previous study in the Haflinger breed, where outcrosses between horses from different countries/stud books were characterised by low S_ROH_ and the lack of ROHs > 6 Mb [15].

All ROH parameters observed in our four subpopulations, reached highest values in the Austrian Lipizzans, whereas the lowest values were found in the Hungarian sample. The values of F_ROH_ generally were in concordance with the findings of pedigree analyses from Zechner et al. [1] and did not exceed 15%. Genomic inbreeding, as described by F_ROH_ in the Lipizzan breed (F_ROH_ = 0.13) was lower than expected from long-term stud farm breeding at small census and it is comparable to values found in the Austrian Haflinger (0.13), the Slovenian Haflinger (0.12) and the Bosnian Mountain Horse (0.14) [15, 16]. Higher levels of F_ROH_ were identified in Shagya Arabians (F_ROH_ = 0.16) and Purebred Arabians (F_ROH_ = 0.18), two breeds characterized by early closure of stud books [15].The length of ROHs is expected to correlate to ancient and recent inbreeding due to number of recombination events. Thus, recent inbreeding events result in longer ROHs as only a few identical by descent (IBD) segments can be broken down by repeated meiosis. According to Browning and Browning [17] and Thompson [18] ROHs of a length of 16.6, 10.0 and 5.0 Mb are assumed to origin from common ancestors back in the 3rd, 5th and 10th generations (6, 10 and 20 meioses respectively). Considering a mean generation interval of 10 years in Lipizzan horses the origin of ROHs in length class of < 2 Mb can be dated to the foundation period of this breed (foundation time 1580 AC). We observed F_ROH_ values calculated based upon ROHs shorter than 2 Mb ranging from 5.6 to 7.1%. F_ROH > 10Mb_ was absent in Slovakian Lipizzans and tended toward zero in Croatian and Hungarian horses, indicating minimal recent inbreeding within the past five generations [18, 19]. In the Austrian population F_ROH > 10Mb_ reached 1.1%. These results demonstrated that it is feasible to minimize the increase of inbreeding in populations limited in size and closed stud books. The ROH profile illustration for the Austrian Lipizzan revealed a strong right shift and a higher variance typical for consanguineous and bottlenecked populations according to Ceballos et al. [20]. The Slovakian sample was characterized by a similar profile, but at a much lower extend, whereas the Croatian and Hungarian samples showed the effects of small census [20].

The identification of overlapping homozygous regions confirmed a closer genetic relationship between the Hungarian and Croatian samples as well as between the Austrian and Slovakian samples, which was also highlighted within the high-resolution population network. Private islands were detected for the Hungarian sample on ECA8, ECA14, ECA23 and ECA30 and for the Austrian sample on ECA3, ECA5, ECA8, ECA16 and ECA20. Within the Austrian Lipizzans an overlapping 953.3kb long homozygous region on ECA16:37.87–38.82 Mb was found, that contained among 29 annotated genes the two genes *ECATH-3* and *ECATH-2.* Both were described as functional genes encoding Cathelicidin-derived antimicrobial peptides, involved in the peptide-based host defence of neutrophils and epithelia in horses [21, 22]. GO analysis for the Austrian Lipizzan gene list highlighted the term GO:0006950~response to stress for the gene *UCN2* (urocortin 2, a member of the corticotropin-releasing hormone family) on ECA16 and the gene *HSP90AB1* (*Equus caballus* heat shock protein 90 alpha family class B member 1) on ECA20, whereas the latter was assigned to four *Equus caballus* species by Vidale et al. [23]. *HSP90AB1* is considered to play a role in the age-dependent metabolism of chondrocytes in horses [24].

All subpopulations shared a homozygous region on ECA11, nearly identical in position and length (mean length 676.4 kb) at 24.13–24.81 Mb containing the annotated genes *COPZ2, CBX1, SNX11, HOXB1, HOXB2, HOXB3, HOXB5, HOXB6, HOXB7, HOXB8, HOXB13, TTLL6* (and *CALCOCO2,* additional in the Hungarian sample). For the Croatian/Hungarian Lipizzans an additional island on ECA4:58.10–58.60 Mb was confirmed containing the homeobox-A cluster. Enrichment analysis highlighted for all samples highly significant levels (*p* < 0.001) for the homeobox-B cluster, mostly related to biological processes involved in embryonic morphogenesis and anterior/posterior specification (GO:0009952, GO:0048704, GO:0009953, GO:0009954). Significant levels for GO terms based upon the HOXA cluster (GO:0060065~uterus development, GO:0009954~proximal/distal pattern formation, GO:0007338~single fertilization, GO:0008584~male gonad development) were exclusively documented for Croatian and Hungarian Lipizzans. The island on ECA11 including the *HOXB* cluster and highly significant GO terms related to morphogenesis were recently also documented in the Posavina horse by Grilz-Seger et al. [16]. Zhang et al. [25] listed *HOXB13* in a recent selection signature study in horses. *HOX* genes play a fundamental role for morphological diversity in animals and for the control of axial morphology along the anterior-posterior body axis. As these gene clusters are directly assigned to axial body segments in numerical order, they can control single segments during embryogenesis concerning their position, segmentation and further differentiation [26].

On ECA11:31.01–31.94 another common homozygous region was found in all samples, whereas the frequency in Hungarian Lipizzans was with 47% slightly below the threshold. This ROH island, nearly identical in length (926.9/910.2 kb) in the Austrian and Croatian Lipizzans, harboured the genes *C11H117orf67, DGKE, COIL, SCPEP1, AKAP1, MSI2* and was partially overlapping with two smaller islands of the Slovakian population. The Hungarian sample had a 244.4 long overlapping homozygous region within the Musashi RNA binding protein 2 (*MSI2*), which is involved in stem cell proliferation [27], haematopoiesis and can promote aggressive myeloid leukaemia in humans [28]. Four of these genes (*COIL, SCPEP1, AKAP1, MSI2*) were listed among other 121 genes that underwent positive selection during the domestication process of the horse [29].

In general the majority of ROH islands were medium sized (between 100 and 900 kb) to small sized (8–100 kb) according to the classification of Pemperton et al. [30]. Only in the Croatian sample one island on ECA14 was longer than 1 Mb. Most of the short ROH islands did not exceed the length of the contained gene, e.g. the island on ECA3:118.66–118.76 Mb, containing the two genes UV-stimulated scaffold protein A (*UVSSA)* and macrophage erythroblast attacher (*MAEA*). *UVSSA* is involved into the repair process of DNA damaged by ultraviolet (UV) sun rays. These two genes were directly followed by a 83 kb long island containing the gene C-terminal-binding protein 1 (*CTBP1*), a transcriptional co-repressor. Pemperton et al. [30] emphasized the investigation of short ROHs (< 100 kb), as they reflect homozygosity for ancient haplotypes contributing to local linkage disequilibrium (LD) patterns. Intermediary ROHs longer than 100 kb up to 2.5 Mb are deemed to be the result from background relatedness in populations with limited census. Furthermore, Pemperton et al. [30] stated that by the commonly applied windows approach as implemented in PLINK [31], ancient haplotypes and small ROHs due to high recombination rate might remain undetected. With the 500 kb applied boundary we were able to detect very small ROH islands, but SNP extraction revealed, that the frequencies of animals sharing those islands were underestimated, exemplarily demonstrated for the genes *MAEA*, *UVSSA*, *CTBP1* and the *STX17* locus.

Grey coat color, caused by a 4.6 kb duplication in intron 6 of Syntaxin 17 (*STX17*), is an essential part of the breeding objective in Lipizzan horses and it has been under selection since the past 150 years. The *STX17* mutation is embedded into 352 kb long haplotype [13, 32].The frequency of the identified ROH island (ECA25:6,39-6,79) harbouring the genes *STX17*, *NR4A3*, *ERP44* and *INVS*, was in concordance with genotype frequencies cited in Pielberg et al. [13], and ranged from 51.1% (Croatia) to 71.2% (Austria) among the respective subpopulations. In the Austrian breeding program only grey horses are used for reproduction, whilst the Slovakian, Hungarian and Croatian stud farms also permit solid colored horses. As a consequence the proportion of homozygous grey *G/G* horses is higher in the Austrian population.

The allele status of *STX17* influences the progression of greying by age, grade of speckling (pigmentation among grey hair), grade of vitiligo and incidences and grade of melanoma [10–13]. The incidence of melanoma in Lipizzans was reported to affect 80% of the animals older than 15 years [10, 13]. Mostly melanomas in Lipizzans are benign and do not metastasize [33]. A previous study revealed, that the genes *STX17*, *NR4A3*, *ERP44* and *INVS,* which are located in the aforementioned ROH island also were expressed in melanoma tissues [13]. Pielberg et al. [13] observed an over-expression of *NR4A3* (nuclear receptor subfamily 4, group A, member 3), which is influenced by the dominant G-allele. The underlying molecular processes are not clearly understood [34]. Seltenhammer et al. [33] investigated factors retarding metastasis in affected grey Lipizzan horses and revealed common features with blue nevi in humans and identified potential markers for equine melanoma (S-100, PCNA, HMB-45, Ki-67, T-311, CD44). We identified within the entire Lipizzan sample an island on ECA14 containing the genes *SPRY4* (Sprouty4) and *NDFI1* (Nedd4-family interacting protein 1). *SPYR4* is a member of the SPYR proteins, which are negative regulators of growth factor signaling pathways. Thus, *SPRY4* inhibits transformed cell growth, migration and invasion, and epithelial-mesenchymal transition [35]. Shaverdashvili et al. [36] provided evidence in human melanoma for an inverse relationship between the expression of *SPYR4* and Membrane-type 1 Matrix Metalloproteinase (*MT1-MMP*), one important driver of melanoma metastasis. Inhibition of *MT1-MMP* resulted in increased expression of *SPYR4* and was correlated with the survival of melanoma patients in humans. *NDFIP1* also acts as a tumour suppressor by repressing cell proliferation and was reported to be down-regulated in human uveal melamoma [37]. In a previous study Jiang et al. [34] investigated the ERK pathway and studied the effects of *BRAF, RAS, GNAQ, GNA11, KIT* on melanoma development in grey horses, whereas an association between *STX17* and an activation of the ERK pathway was shown, but no activation of the aforementioned genes were observed. In the Austrian Lipizzan sample we identified an island containing the gene *HSP90AB1*. Metri et al. [38] identified *HSP90AB1* as a main discriminator between metastatic and primary melanoma in humans, which also was correlated with survival rate of melanoma patients. In our data GO analysis revealed an enrichment of *HSP90AB1* in the terms GO:0069550-reponse to stress and GO:0071353-cellular response to interleukin-4.

## Conclusions

The enhanced high-resolution Netview approach results in a generally understandable visualisation, which is able to simultaneously present fundamental metrics of genetic diversity, coancestry and relatedness on population and individual level. This approach thus provides a high practical impact for conventional and conservation breeding management. The analyses of ROH islands based upon the conventional ROH size boundary of > = 500 kb have proven to be a valuable approach to screen for signatures of selection, also able to detect short homozygous overlapping regions. In order to avoid an underestimation of the frequencies of very short ROHs the underlying size boundary should be adapted in regions of special interest. The investigation of subpopulations within one single breed allows a fine-scale analysis and we demonstrated that slightly different breeding objectives have a high impact on genomic regions and gene content. ROH island analyses identified several annotated genes (*SPRY4*, *NDFIP1*, *HSP90AB1 and IMPDH2*), which may play a role for further studies on equine melanoma.

## Methods

### Ethics statement

This study was discussed and approved by the institutional Commission for Ethics and Animal Welfare, University of Veterinary Medicine, Vienna, protocol number: ETK-06/05/2015, in accordance with GSP guidelines and national legislation. The Lipizzan state stud farms Piber (Austria), Topol’čianky (Slovakia), Lipik, Đakovo (Croatia) and Szilvasvárad (Hungary) granted the permission to take hair samples from their horses.

### Sampling

The horses included in this study originated from five European state stud farms, which are located in four different countries. The Austrian and the Slovakian samples represent the entire breeding populations of the stud farm of Piber (Austria, 254 horses) and Topol’čianky (Slovakia, 55 horses). The Croatian Lipizzans were sampled from the state stud farms Lipik (8 horses) and Đakovo (37 horses), whilst 23 Lipizzan horses were collected from the Hungarian state stud farm Szilvasvárad.

The hair samples were taken between the years 2013 and 2017 and the selected horses were born between 1989 and 2014. For Austria and Slovakia we were able to collect the whole stud farm populations. The breeding stock of the Croatian stud farms comprises together approximately 120 breeding animals, the number in the Hungarian state stud farm is on a similar level.

### SNP genotyping

The single nucleotide polymorphism (SNP) genotypes for the 377 horses were determined using the Affymetrix Axiom™ Equine genotyping array [14]. The chromosomal position of the SNPs was derived from EquCab2.0 reference genome. We excluded SNPs positioned on the sex chromosomes (X: 28,017 SNPs and Y: 1 SNP), SNPs without known chromosomal position (30,864 SNPs) and SNPs with more than 10% missing genotypes, resulting in a total of 611,914 SNPs for each horse. For the analysis of population structure and genetic diversity we excluded SNPs with a minor allele frequency (MAF) less than 0.01, resulting in 492,719 SNPs per horse.

### Genetic diversity and ROH analysis

In order to illustrate the population structure we applied Principal Component Analysis (PCA) on basis of the genetic relationship matrix (G) with pairwise identities by state (IBS) between horses as provided by PLINK v.1.7 [31]. The PCA was performed using R platform, whilst pairwise F_ST_ values were calculated with the R package Geneland (https://rdrr.io/cran/Geneland).

Population clustering and admixture analysis were determined using the program Admixture 1.23 [39]. We ran Admixture for 100 iterations increasing K from 2 to 10. Convergence between independent runs at the same K was monitored by comparing the resulting log-likelihood scores (LLs) following 100 iterations, and was inferred from stabilized LLs with less than 1 LL unit of variation between runs. Cross validation (CV) error estimation for each K was performed to determine the optimal number of clusters. Admixture results were visualized with the program Distruct 1.1 [40] and integrated in the high-resolution network following the procedure of Druml et al. [15].

ROH were determined with an overlapping window approach implemented in PLINK v1.7 [31] based on the settings: minimum SNP density = one SNP per 50 kb; maximum gap length = 100 kb; minimum length of homozygous segment > 500 kb (including more than 80 homozygous SNPs); one heterozygote and two missing genotypes were permitted within each segment.

The total number of ROH (N_ROH_), average length of ROH (L_ROH_) and the sum of all ROH segments (S_ROH_) were summarized according to respective subpopulations. To analyze ROH length classes, ROHs were divided into following seven length categories: 0.5–1, 1–2, > 2–4, > 4–6, > 6–8, > 8–10 and > 10 Mb. The genomic inbreeding coefficients (F_ROH_) were calculated by use of following formular:

$$ {F}_{ROH}=\sum \frac{L_{ROH}}{L_{AUTO}} $$

,whereas the length of the autosomal genome (L_AUTO_), was set to 2243 GB. Statistical analyses and graphical representations of specific genomic regions and distribution of ROH segments were performed using the R-package detectROHs (www.r-project.org)and SAS v.9.1. [41].

### High-resolution population networks

In order to ascertain the high-resolution population structure of Lipizzan horses, we performed a network visualization based upon the aforementioned IBS derived relationship matrix (G), following the description of Druml et al. [15]. The different components involved in the so-called NetView approach are described in detail by Neuditschko et al. [42] and Steining et al. [43]. Briefly, we computed genetic distances by subtracting pairwise relationships from 1 and applied the algorithm in its default setting (number of *k* nearest neighbors *k*-NN = 10). To illustrate the genetic relatedness between neighboring horses, we associated the thickness of edges (connecting lines) with the proportion of the genetic distance, whilst thicker edges corresponding to lower genetic distances. To identify highly inbred and outcrossed horses within the respective population networks we scaled the node size of each horse based on the individual S_ROH_. The node colors of each horse represent the individual level of admixture at the selected number of K-clusters, whilst the node border colors illustrate the origin of the horses.

### Gene characterization in ROH islands

The distribution of ROH segments across the genome was visualized using the R-package detect ROHs (www.r-project.org). Putative ROH islands were determined based upon overlapping homozygous regions, which were shared by more than 50% of the horses. ROH islands were calculated for the entire data sample and respective subpopulations. Genes located in ROH regions were identified with the map viewer of the equine ensemble database EquCab2.0 (www.ensembl.org) and Gene Ontology (GO) terms and KEGG (Kyoto Encyclopedia of Genes and Genomes) pathways of annotated genes were analyzed using the open source Database for Annotation, Visualization, and Integrated Discovery (DAVID) v6.8 package (https://david.ncifcrf.gov) using the *Equus caballus* annotation file as background [44].

## Additional files

Additional file 1: Pairwise F_ST_ values of the four Lipizzan subpopulations (Austrian, Croatia, Hungary, Slovakia). (DOC 41 kb)

Additional file 2: Graphical representation of Cross validation error to determine an optimal number of K clusters. (JPG 454 kb)

Additional file 3: Profile plot of S_ROH_ versus N_ROH_. Individuals are illustrated as dots (Đakovo/Croatia = blue; Lipik/Croatia = red; Piber/Austria = green; Szilvasvárad/Hungary = brown; Topol’čianky/Slovakia = purple). (DOCX 191 kb)

Additional file 4: Gene Ontology (GO) terms and KEGG pathways based on annotated genes embedded in ROH islands for the entire Lipizzan sample. (DOC 44 kb)

Additional file 5: Gene Ontology (GO) terms and KEGG pathways based on annotated genes embedded in ROH islands for the Lipizzan horses from the Austrian stud farm Piber. (DOC 47 kb)

Additional file 6: Gene Ontology (GO) terms and KEGG pathways based on annotated genes embedded in ROH islands for the Lipizzan horses from the Slovak national stud farm of Topol’čianky. (DOC 48 kb)

Additional file 7: Gene Ontology (GO) terms and KEGG pathways based on annotated genes embedded in ROH islands for the Lipizzan horses from the Croatian state stud farms Lipik/Đakovo. (DOC 61 kb)

Additional file 8: Gene Ontology (GO) terms and KEGG pathways based on annotated genes embedded in ROH islands for the Lipizzan horses from the Hungarian state stud farm Szilvasvárad. (DOC 62 kb)

## Abbreviations

*ADRM1*: *Proteasomal ubiquitin receptor ADRM1*
*AKAP1*: *A-kinase anchoring protein 1*
*ALS2CL*: *ALS2 C-terminal-like protein*
*ARHGAP26*: *Rho GTPase activating protein 26*
*ARHGEF4*: *Rho guanine nucleotide exchange factor 4*
*ARIH2*: *Ariadne RBR E3 ubiquitin protein ligase 2*
*ATRIP*: *ATR interacting protein*
*B3GNT4*: *UDP-GlcNAc:betaGal beta-1,3-N-acetylglucosaminyltransferase 4*
*BCL7A*: *BAF chromatin remodeling complex subunit BCL7A*
bp: Base pair
*BRAF*: *B-Raf proto-oncogene, serine/threonine kinase*
*C11H17orf67*: *Chromosome 11 C17orf67 homolog*
*C16H3orf84*: *Chromosome 16 H17orf84 homolog*
*CABLES2*: *CDK5 and ABL1 enzyme substrate 2*
*CAPN11*: *Calpain-11*
*CBX1*: *Chromobox 1*
*CCDC12*: *Coiled-coil domain-containing protein 12*
*CCDC51*: *Coiled-coil domain containing 51*
*CCDC71*: *Coiled-coil domain containing 71*
*CDH*: *Cadherin*
*CELSR3*: *Cadherin EGF LAG seven-pass G-type receptor 3*
*CHD1L*: *Chromodomain Helicase DNA Binding Protein 1 Like*
*CLIP1*: *CAP-Gly domain containing linker protein 1*
*COIL*: *Coilin*
*COPZ2*: *Coatomer Protein Complex Subunit Zeta 2*
*CTBP1*: *C-Terminal Binding Protein 1*
*CTDP1*: *CTD Phosphatase Subunit 1*
CV: Cross validation
*DALRD3*: *DALR anticodon binding domain containing 3*
DAVID: Database for Annotation, Visualization and Integrated Discovery
*DELE1*: *DAP3 binding cell death enhancer 1*
*DGKE*: *Diacylglycerol kinase epsilon*
*DIABLO*: *Diablo IAP-binding mitochondrial protein*
ECA11: Equine chromosome 11
ECA14: Equine chromosome 14
ECA16: Equine chromosome 16
ECA18: Equine chromosome 18
ECA20: Equine chromosome 20
ECA25: Equine chromosome 25
ECA3: Equine chromosome 3
ECA30: Equine chromosome 30
ECA5: Equine chromosome 5
ECA8: Equine chromosome 8
*ECATH-2*: *Myeloid cathelicidin 2*
*ECATH-3*: *Myeloid cathelicidin 3*
*EDN3*: *Endothelin 3*
EquCab2.0: *Equus caballus reference genome version 2*
ERK pathway: Extracellular-signal-regulated kinase
*ERP44*: *Endoplasmic reticulum protein 44*
*EVX1*: *Even-skipped homeobox 1*
*FGF1*: *Fibroblast growth factor 1*
*FMO5*: *Flavin Containing Monooxygenase 5*
F_ROH_: Inbreeding coefficient based upon ROH
F_ST_: Fixation coefficient
*FSTL4*: *Follistatin like 4*
G: Genetic relationship matrix
*GATA5*: *Transcription factor GATA-5*
*GLDC*: *Glycine decarboxylase*
*GNA1*: *Glucosamine 6-phosphate N-acetyltransferase*
*GNAQ*: *G protein subunit alpha q*
*GNPDA1*: *Glucosamine-6-phosphate deaminase 1*
GO: Gene ontology
*HIBADH*: *3-hydroxyisobutyrate dehydrogenase, mitochondrial*
*HOXA1*: *Homeobox A1*
*HOXA10*: *Homeobox A10*
*HOXA11*: *Homeobox A11*
*HOXA2*: *Homeobox A2*
*HOXA3*: *Homeobox A3*
*HOXA5*: *Homeobox A5*
*HOXA6*: *Homeobox A6*
*HOXA7*: *Homeobox A7*
*HOXA9*: *Homeobox A9*
*HOXB*: *Homeobox B*
*HOXB1*: *Homeobox B1*
*HOXB13*: *Homeobox B13*
*HOXB2*: *Homeobox B2*
*HOXB3*: *Homeobox B3*
*HOXB5*: *Homeobox B5*
*HOXB6*: *Homeobox B6*
*HOXB7*: *Homeobox B7*
*HOXB8*: *Homeobox B8*
*HPD*: *4-hydroxyphenylpyruvic acid dioxygenase*
*HRH3*: *Histamine H3 receptor*
*HSP90AB1*: *Equus caballus heat shock protein 90 alpha family class B member 1*
IBS: Identity by state
*IL31*: *Interleukin 31*
*IMPDH2*: *Inosine monophosphate dehydrogenase 2*
*INVS*: *Inversin*
*IP6K2*: *Inositol hexakisphosphate kinase 2*
K: Cluster
kb: Kilobase
*KDM4C*: *Lysine demethylase 4C*
KEGG: Kyoto Encyclopedia of Genes and Genomes
*KIF26B*: *Kinesin family member 26B*
*KIT*: *KIT proto-oncogene, receptor tyrosine kinase*
*KLHDC8B*: *Kelch domain containing 8B*
*LAMA5*: *Laminin subunit alpha-5*
*LAMB2*: *Laminin subunit beta 2*
LD: Linkage disequilibrium
L_ROH_: ROH length
*LRRC43*: *Leucine rich repeat containing 43*
*LSM14B*: *LSM family member 14B*
*MAEA*: *Macrophage Erythroblast Attacher*
MAF: Minor allele frequency
Mb: Megabase
*MLXIP*: *MLX interacting protein*
*MSI2*: *Musashi RNA binding protein 2*
mtDNA: Mitochondrial deoxyribonucleic acid
*MTG2*: *Mitochondrial ribosome-associated GTPase 2*
*NCKIPSD*: *NCK interacting protein with SH3 domainM*
*NDFIP1*: *Nedd4 family interacting protein 1*
*NDUFAF3*: *NADH:Ubiquinone oxidoreductase complex assembly factor 3*
*NFKBIE*: *NF-kappa-B inhibitor epsilon*
*NME6*: *NME/NM23 nucleoside diphosphate kinase 6*
*NR4A3*: *Nuclear receptor subfamily 4 group A member 3*
N_ROH_: mean number of ROHs
OMIA: Online Mendelian Inheritance in Animals
OMIM: Online Mendelian Inheritance in Man
*OSBPL2*: *Oxysterol-binding protein-related protein 2*
*P4HTM*: *Prolyl 4-hydroxylase, Transmembrane*
PC: Principal component
PCA: Principal component analysis
*PCDH1*: *Protocadherin 1*
*PCDH12*: *Protocadherin 12*
*PFKFB4*: *6-Phosphofructo-2-kinase/fructose-2,6-biphosphatase 4*
*PHACTR3*: *Phosphatase and actin regulator 3*
*PLEKHB2*: *Pleckstrin homology domain-containing family B member 2*
*PLXNB1*: *Plexin B1*
*PRKAB2*: *Protein kinase AMP-activated non-catalytic subunit beta 2*
*PSMA7*: *Proteasome subunit alpha type-7*
*PSMD9*: *Proteasome 26S subunit, non-ATPase 9*
*QARS*: *Glutaminyl-TRNA synthetase*
*QRICH1*: *Glutamine rich 1*
*RAS*: *Resistance to audiogenic seizures*
*RBBP8NL*: *RBBP8 N-terminal-like protein*
*RHOF*: *Ras homolog family member F, filopodia associated*
*RNF14*: *Ring finger protein 14*
ROH: Runs of homozygosity
*SCPEP1*: *Serine carboxypeptidase 1*
SD: Standard deviation
*SETD1B*: *SET domain containing 1B, histone lysine methyltransferase*
*SETD2*: *Histone-lysine N-methyltransferase SETD2*
*SHISA5*: *Shisa family member 5*
*SLC26A6*: *Solute carrier family 26 member 6*
*SLC29A1*: *Solute carrier family 29 member 1*
*SLC35B2*: *Solute carrier family 35 member B2*
*SMYD3*: *SET and MYND domain containing 3*
SNP: Single nucleotide polymorphism
*SNX11*: *Sorting nexin 11*
*SPRY4*: *Sprouty4*
S_ROH_: mean genome coverage of ROH
*SS18L1*: *SS18L1 subunit of BAF chromatin remodeling complex*
*STX17*: *Syntaxin 17*
*TAF4*: *Transcription initiation factor TFIID subunit 4*
*TCTE1*: *T-complex-associated-testis-expressed 1*
*TMEM120B*: *Transmembrane protein 120B*
*TMEM89*: *Transmembrane protein 89*
*TMIE*: *Transmembrane inner ear expressed protein*
*TREX1*: *Three prime repair exonuclease 1*
*TTLL6*: *Tubulin tyrosine ligase like 6*
*UCN2*: *Urocortin 2*
*UHRF2*: *Ubiquitin like with PHD and ring finger domains 2*
*UQCRC1*: *Ubiquinol-cytochrome c reductase core protein 1*
*USP19*: *Ubiquitin specific peptidase 19*
UV: ultraviolet
*UVSSA*: *UV Stimulated Scaffold Protein A*
*WDR6*: *WD repeat domain 6*
*WDR66*: *WD repeat domain 66*
*ZNF831*: *Zinc finger protein 831*
