# Supplementary material for: High-resolution population structure and runs of homozygosity reveal the genetic architecture of complex traits in the Lipizzan horse
Source: BMC Genomics. 2019 Mar 5;20:174. doi: 10.1186/s12864-019-5564-x (PMC6402180; doi:10.1186/s12864-019-5564-x)
Supplement: Supplementary file 1 — Pairwise FST values of the four Lipizzan subpopulations (Austrian, Croatia, Hungary, Slovakia). (DOC 41 kb) [file 12864_2019_5564_MOESM1_ESM.doc]

**Additional File 1** Pairwise FST values based upon xxxxxx SNPs in the four Lipizzan subpopulations (Austrian, Croatia, Hungary, Slovakia)

|  | **Austria** | **Croatia** | **Slovakia** |
| --- | --- | --- | --- |
| **Croatia** | 0.045 |  |  |
| **Slovakia** | 0.043 | 0.025 |  |
| **Hungary** | 0.092 | 0.080 | 0.068 |
