# Supplementary figures and images for: High-resolution population structure and runs of homozygosity reveal the genetic architecture of complex traits in the Lipizzan horse
Source: BMC Genomics. 2019 Mar 5;20:174. doi: 10.1186/s12864-019-5564-x (PMC6402180; doi:10.1186/s12864-019-5564-x)

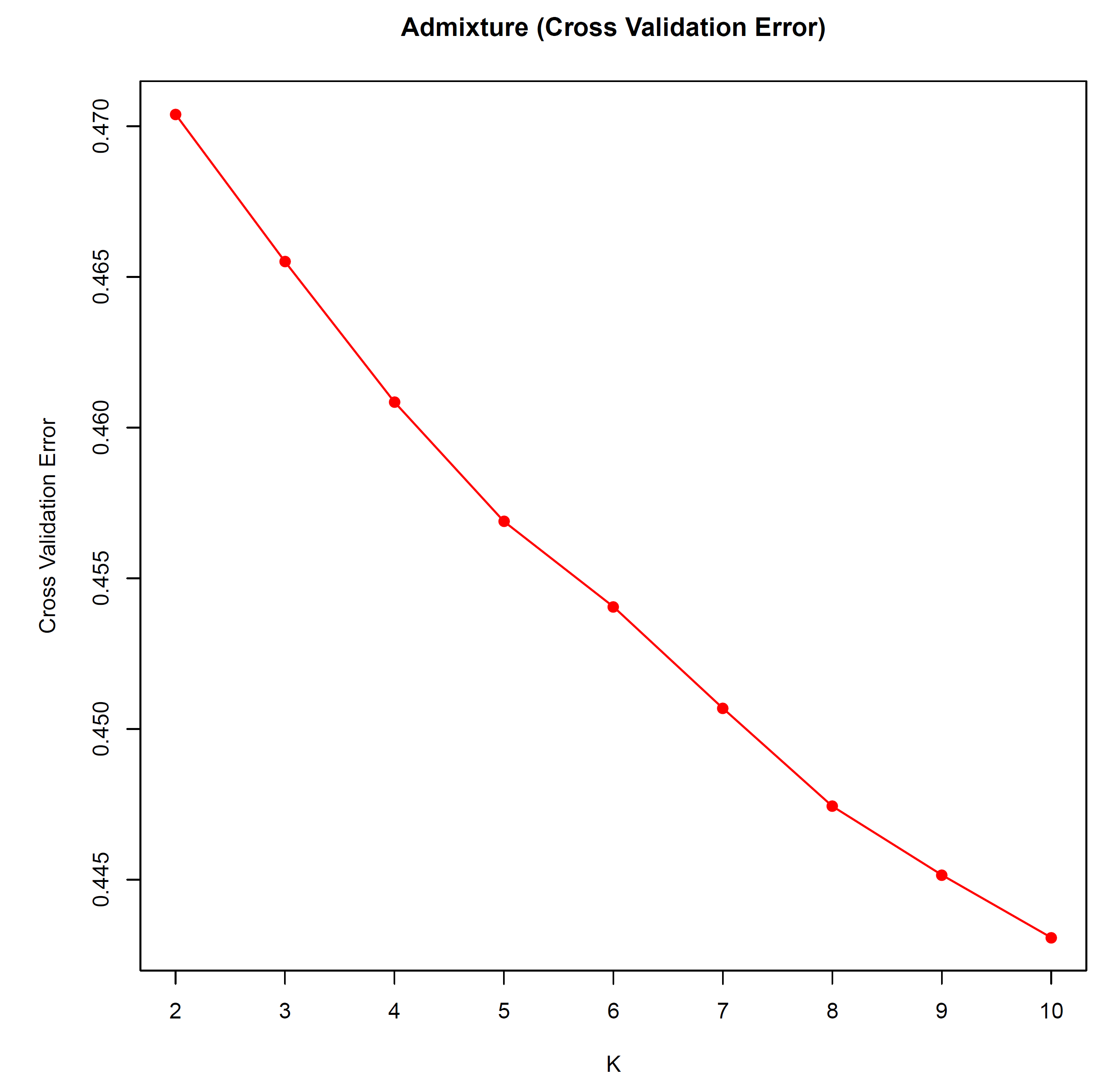

Supplement: Supplementary file 2 — Graphical representation of Cross validation error to determine an optimal number of K clusters. (JPG 454 kb) [file 12864_2019_5564_MOESM2_ESM.jpg]
