# Supplementary material for: High-resolution population structure and runs of homozygosity reveal the genetic architecture of complex traits in the Lipizzan horse
Source: BMC Genomics. 2019 Mar 5;20:174. doi: 10.1186/s12864-019-5564-x (PMC6402180; doi:10.1186/s12864-019-5564-x)

**Additional File 3** Profile plot of S_ROH_ versus N_ROH_. Individuals are illustrated as dots (Đakovo/Croatia = blue; Lipik/Croatia = red; Piber/Austria = green; Szilvasvárad/Hungary = brown; Topol’čianky/Slovakia = purple).


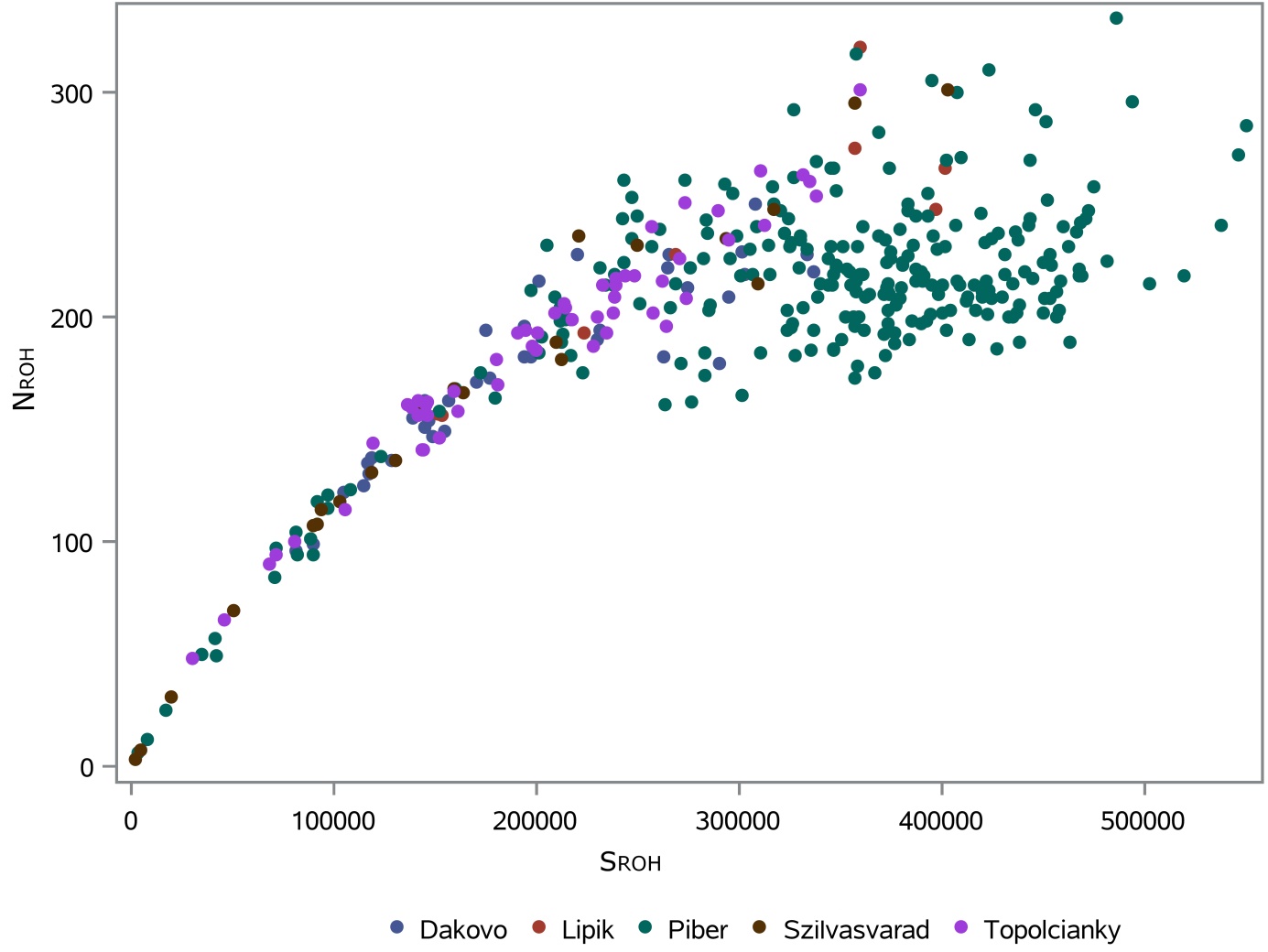

Supplement: Supplementary file 3 — Profile plot of SROH versus NROH. Individuals are illustrated as dots (Đakovo/Croatia = blue; Lipik/Croatia = red; Piber/Austria = green; Szilvasvárad/Hungary = brown; Topol’čianky/Slovakia = purple). (DOCX 191 kb) [file 12864_2019_5564_MOESM3_ESM.docx]
