# Supplementary material for: High-resolution population structure and runs of homozygosity reveal the genetic architecture of complex traits in the Lipizzan horse
Source: BMC Genomics. 2019 Mar 5;20:174. doi: 10.1186/s12864-019-5564-x (PMC6402180; doi:10.1186/s12864-019-5564-x)
Supplement: Supplementary file 4 — Gene Ontology (GO) terms and KEGG pathways based on annotated genes embedded in ROH islands for the entire Lipizzan sample. (DOC 44 kb) [file 12864_2019_5564_MOESM4_ESM.doc]

**Additional File 4** Gene Ontology (GO) terms and KEGG pathways based on annotated genes embedded in ROH islands for the entire Lipizzan sample

| **Term** | **p-value** | **Genes** | **Fold Enrichment** | **Bonferroni adjusted p-value** |
| --- | --- | --- | --- | --- |
| ***Biological process*** |  |  |  |  |
| GO:0048704~embryonic skeletal system morphogenesis | <0.001 | *HOXB3, HOXB1, HOXB2, HOXB7, HOXB8, HOXB5, HOXB6* | 159.70 | <0.001 |
| GO:0009952~anterior/posterior pattern specification | <0.001 | *HOXB3, HOXB1, HOXB2, HOXB7, HOXB8, HOXB5, HOXB6* | 73.89 | <0.001 |
| GO:0021570~rhombomere 4 development | 0.003 | *HOXB1, HOXB2* | 707.24 | 0.134 |
| GO:0021612~facial nerve structural organization | 0.011 | *HOXB1, HOXB2* | 176.81 | 0.438 |
| ***Molecular function*** |  |  |  |  |
| GO:0043565~sequence-specific DNA binding | <0.001 | *HOXB1, HOXB2, HOXB7, HOXB6, HOXB13* | 16.37 | 0.002 |
| GO:0003700~transcription factor activity, sequence-specific DNA binding | 0.007 | *HOXB2, HOXB7, HOXB8, HOXB6* | 9.10 | 0.105 |
