# Supplementary material for: High-resolution population structure and runs of homozygosity reveal the genetic architecture of complex traits in the Lipizzan horse
Source: BMC Genomics. 2019 Mar 5;20:174. doi: 10.1186/s12864-019-5564-x (PMC6402180; doi:10.1186/s12864-019-5564-x)
Supplement: Supplementary file 5 — Gene Ontology (GO) terms and KEGG pathways based on annotated genes embedded in ROH islands for the Lipizzan horses from the Austrian stud farm Piber. (DOC 47 kb) [file 12864_2019_5564_MOESM5_ESM.doc]

**Additional File 5** Gene Ontology (GO) terms and KEGG pathways based on annotated genes embedded in ROH islands for the Lipizzan horses from the Austrian stud farm Piber

| **Term** | **p-value** | **Genes** | **Fold Enrichment** | **Bonferroni adjusted p-value** |
| --- | --- | --- | --- | --- |
| ***Biological process*** |  |  |  |  |
| GO:0048704~embryonic skeletal system morphogenesis | <0.001 | *HOXB3, HOXB1, HOXB2, HOXB7, HOXB8, HOXB5, HOXB6* | 53.63 | <0.001 |
| GO:0009952~anterior/posterior pattern specification | <0.001 | *HOXB3, HOXB1, HOXB2, HOXB7, HOXB8, HOXB5, HOXB6* | 24.63 | <0.001 |
| GO:0021570~rhombomere 4 development | 0.009 | *HOXB1, HOXB2* | 235.75 | 0.762 |
| GO:0021612~facial nerve structural organization | 0,033 | *HOXB1, HOXB2* | 58,94 | 0,997 |
| GO:0006183~GTP biosynthetic process | 0,033 | *IMPDH2, NME6* | 58,94 | 0,997 |
| GO:0006950~response to stress | 0,057 | *HSP90AB1, UCN2* | 33,68 | 0,999 |
| GO:0071353~cellular response to interleukin-4 | 0,068 | *HSP90AB1, IMPDH2* | 27,73 | 0,999 |
| ***Molecular function*** |  |  |  |  |
| GO:0043565~sequence-specific DNA binding | 0,012 | *HOXB1, HOXB2, HOXB7, HOXB6, HOXB13* | 5,46 | 0,546 |
| GO:0003700~transcription factor activity, sequence-specific DNA binding | 0,039 | *CTBP1, HOXB2, HOXB7, HOXB8, HOXB6* | 3,79 | 0,927 |
