# Supplementary material for: High-resolution population structure and runs of homozygosity reveal the genetic architecture of complex traits in the Lipizzan horse
Source: BMC Genomics. 2019 Mar 5;20:174. doi: 10.1186/s12864-019-5564-x (PMC6402180; doi:10.1186/s12864-019-5564-x)
Supplement: Supplementary file 6 — Gene Ontology (GO) terms and KEGG pathways based on annotated genes embedded in ROH islands for the Lipizzan horses from the Slovak national stud farm of Topol’čianky. (DOC 48 kb) [file 12864_2019_5564_MOESM6_ESM.doc]

**Additional File 6** Gene Ontology (GO) terms and KEGG pathways based on annotated genes embedded in ROH islands for the Lipizzan horses from the Slovak stud farm of Topol’čianky

| **Term** | **p-value** | **Genes** | **Fold Enrichment** | **Bonferroni adjusted p-value** |
| --- | --- | --- | --- | --- |
| ***Biological process*** |  |  |  |  |
| GO:0048704~embryonic skeletal system morphogenesis | <0.001 | *HOXB3, HOXB1, HOXB2, HOXB7, HOXB8, HOXB5, HOXB6* | 93.62 | <0.001 |
| GO:0009952~anterior/posterior pattern specification | <0.001 | *HOXB3, HOXB1, HOXB2, HOXB7, HOXB8, HOXB5, HOXB6* | 43.31 | <0.001 |
| GO:0021570~rhombomere 4 development | 0.005 | *HOXB1, HOXB2* | 414.59 | 0.455 |
| GO:0048864~stem cell development | 0.007 | *MSI2, SETD2* | 276.39 | 0.597 |
| GO:0021612~facial nerve structural organization | 0.019 | *HOXB1, HOXB2* | 103.65 | 0.912 |
| GO:0001525~angiogenesis | 0.021 | *HOXB3, HOXB13, SETD2* | 12.82 | 0.939 |
| GO:0006368~transcription elongation from RNA polymerase II promoter | 0.039 | *ADRM1, SETD2* | 48.77 | 0.994 |
| ***Cellular component*** |  |  |  |  |
| GO:0005634~nucleus | 0.019 | *HOXB3, HOXB1, HOXB2, LAMA5, HOXB8, HOXB5, HOXB6, SETD2, PSMA7, RNF14* | 2.26 | 0.528 |
| ***Molecular function*** |  |  |  |  |
| GO:0043565~sequence-specific DNA binding | 0.001 | *HOXB1, HOXB2, HOXB7, HOXB6, HOXB13* | 9.55 | 0.040 |
| GO:0003700~transcription factor activity, sequence-specific DNA binding | 0.005 | *TAF4, HOXB2, HOXB7, HOXB8, HOXB6* | 6.64 | 0.142 |
