# Supplementary material for: High-resolution population structure and runs of homozygosity reveal the genetic architecture of complex traits in the Lipizzan horse
Source: BMC Genomics. 2019 Mar 5;20:174. doi: 10.1186/s12864-019-5564-x (PMC6402180; doi:10.1186/s12864-019-5564-x)
Supplement: Supplementary file 7 — Gene Ontology (GO) terms and KEGG pathways based on annotated genes embedded in ROH islands for the Lipizzan horses from the Croatian state stud farms Lipik/Đakovo. (DOC 61 kb) [file 12864_2019_5564_MOESM7_ESM.doc]

**Additional File 7** Gene Ontology (GO) terms and KEGG pathways based on annotated genes embedded in ROH islands for the Lipizzan horses from the Croatian state stud farms Lipik/Đakovo

| **Term** | **p-value** | **Genes** | **Fold Enrichment** | **Bonferroni adjusted p-value** |
| --- | --- | --- | --- | --- |
| ***Biological process*** |  |  |  |  |
| GO:0009952~anterior/posterior pattern specification | <0,001 | *HOXA11, HOXB3, HOXA2, HOXB1, HOXA3, HOXB2, HOXB7, HOXA5, HOXB8, HOXB5, HOXA6, HOXB6, HOXA7, HOXA10, HOXA9* | 58,52 | <0,001 |
| GO:0048704~embryonic skeletal system morphogenesis | <0,001 | *HOXB3, HOXB1, HOXA3, HOXB2, HOXB7, HOXA5, HOXB8, HOXA6, HOXB5, HOXA7, HOXB6* | 92,74 | <0,001 |
| GO:0060065~uterus development | 0,001 | *HOXA11, HOXA10, HOXA9* | 71,28 | 0,149 |
| GO:0009953~dorsal/ventral pattern formation | 0,002 | *HOXA2, HOXB2, HOXA11* | 41,27 | 0,389 |
| GO:0009954~proximal/distal pattern formation | 0,002 | *HOXA11, HOXA10, HOXA9* | 41,27 | 0,389 |
| GO:0007338~single fertilization | 0,003 | *HOXA11, HOXA10, HOXA9* | 37,34 | 0,453 |
| GO:0030878~thyroid gland development | 0,003 | *HOXB3, HOXA3, HOXA5* | 34,09 | 0,515 |
| GO:0001525~angiogenesis | 0,006 | *HOXB3, HOXA3, HOXA7, HOXB13* | 10,78 | 0,711 |
| GO:0021570~rhombomere 4 development | 0,008 | *HOXB1, HOXB2* | 261,37 | 0,807 |
| GO:0021615~glossopharyngeal nerve morphogenesis | 0,011 | *HOXB3, HOXA3* | 174,25 | 0,915 |
| GO:0008584~male gonad development | 0,012 | *HOXA11, HOXA10, HOXA9* | 17,42 | 0,933 |
| GO:0021612~facial nerve structural organization | 0,029 | *HOXB1, HOXB2* | 65,34 | 0,999 |
| GO:0060216~definitive hemopoiesis | 0,029 | *HOXB3, HOXA9* | 65,34 | 0,999 |
| GO:0001759~organ induction | 0,037 | *HOXA11, FGF1* | 52,27 | 0,999 |
| GO:0007156~homophilic cell adhesion via plasma membrane adhesion molecules | 0,038 | *PCDH1, PCDH12, CDH4* | 9,56 | 0,999 |
| GO:0045638~negative regulation of myeloid cell differentiation | 0,062 | *HOXB8, HOXA9* | 30,75 | 0,999 |
| GO:0050890~cognition | 0,062 | *HOXA1, HRH3* | 30,75 | 0,999 |
| GO:0035115~embryonic forelimb morphogenesis | 0,090 | *HOXA11, HOXA9* | 20,91 | 1,000 |
| GO:0051216~cartilage development | 0,096 | *HOXB3, HOXA3* | 19,36 | 1,000 |
| ***Cellular component*** |  |  |  |  |
| GO:0005634~nucleus | 0,002 | *HOXA11, PSMA7, HOXB3, HOXA1, HOXB1, HOXB2, HOXA5, LAMA5, HOXB8, HOXB5, HOXA6, HOXB6, HOXA7, HOXA10, HOXA9, RNF14* | 2,25 | 0,098211 |
| GO:0005667~transcription factor complex | 0,012 | *HOXA11, HOXA10, HOXA9, HOXB13* | 8,19 | 0,432675 |
| ***Molecular function*** |  |  |  |  |
| GO:0043565~sequence-specific DNA binding | <0,001 | *HOXA1, HOXB1, HOXA3, HOXB2, EVX1, HOXB7, HOXA6, HOXA11, HOXB6, HOXA9, HOXB13* | 12,01 | <0,001 |
| GO:0003700~transcription factor activity, sequence-specific DNA binding | 0,009 | *TAF4, HOXB2, HOXB7, HOXB8, HOXA6, HOXB6* | 4,55 | 0,315 |
| GO:0000978~RNA polymerase II core promoter proximal region sequence-specific DNA binding | 0,060 | *HOXA2, HOXA5, HOXA7, HOXA10* | 4,35 | 0,931 |
