# Supplementary material for: High-resolution population structure and runs of homozygosity reveal the genetic architecture of complex traits in the Lipizzan horse
Source: BMC Genomics. 2019 Mar 5;20:174. doi: 10.1186/s12864-019-5564-x (PMC6402180; doi:10.1186/s12864-019-5564-x)
Supplement: Supplementary file 8 — Gene Ontology (GO) terms and KEGG pathways based on annotated genes embedded in ROH islands for the Lipizzan horses from the Hungarian state stud farm Szilvasvárad. (DOC 62 kb) [file 12864_2019_5564_MOESM8_ESM.doc]

**Additional File 8** Gene Ontology (GO) terms and KEGG pathways based on annotated genes embedded in ROH islands for the Lipizzan horses from the Hungarian state stud farm Szilvasvárad

| **Term** | **PValue** | **Genes** | **Fold Enrichment** | **Bonferroni adjusted p-value** |
| --- | --- | --- | --- | --- |
| ***Biological process*** |  |  |  |  |
| GO:0009952~anterior/posterior pattern specification | <0,001 | *HOXA11, HOXB3, HOXA2, HOXB1, HOXA3, HOXB2, HOXB7, HOXB8, HOXA5, HOXA6, HOXB6, HOXA7, HOXA10, HOXA9* | 67,90 | <0,001 |
| GO:0048704~embryonic skeletal system morphogenesis | <0,001 | *HOXB3, HOXB1, HOXA3, HOXB2, HOXB7, HOXA5, HOXB8, HOXA6, HOXA7, HOXB6* | 104,82 | <0,001 |
| GO:0060065~uterus development | 0,001 | *HOXA11, HOXA10, HOXA9* | 88,62 | 0,086 |
| GO:0009953~dorsal/ventral pattern formation | 0,001 | *HOXA2, HOXB2, HOXA11* | 51,31 | 0,240 |
| GO:0009954~proximal/distal pattern formation | 0,001 | *HOXA11, HOXA10, HOXA9* | 51,31 | 0,240 |
| GO:0007338~single fertilization | 0,002 | *HOXA11, HOXA10, HOXA9* | 46,42 | 0,285 |
| GO:0030878~thyroid gland development | 0,002 | *HOXB3, HOXA3, HOXA5* | 42,38 | 0,332 |
| GO:0001525~angiogenesis | 0,003 | *HOXB3, HOXA3, HOXA7, HOXB13* | 13,40 | 0,435 |
| GO:0021570~rhombomere 4 development | 0,006 | *HOXB1, HOXB2* | 324,95 | 0,680 |
| GO:0008584~male gonad development | 0,008 | *HOXA11, HOXA10, HOXA9* | 21,66 | 0,781 |
| GO:0021615~glossopharyngeal nerve morphogenesis | 0,010 | *HOXB3, HOXA3* | 216,63 | 0,819 |
| GO:0006355~regulation of transcription, DNA-templated | 0,019 | *HOXA1, ZNF831, HOXA3, HOXA9, HOXB13* | 4,64 | 0,978 |
| GO:0060216~definitive hemopoiesis | 0,023 | *HOXB3, HOXA9* | 81,24 | 0,989 |
| GO:0021612~facial nerve structural organization | 0,023 | *HOXB1, HOXB2* | 81,24 | 0,989 |
| GO:0045638~negative regulation of myeloid cell differentiation | 0,049 | *HOXB8, HOXA9* | 38,23 | 0,999 |
| GO:0001578~microtubule bundle formation | 0,061 | *CLIP1, TTLL6* | 30,95 | 0,999 |
| GO:0007283~spermatogenesis | 0,069 | *HOXA11, HOXA10, HOXA9* | 6,72 | 0,999 |
| GO:0035115~embryonic forelimb morphogenesis | 0,072 | *HOXA11, HOXA9* | 26,00 | 0,999 |
| GO:0051216~cartilage development | 0,078 | *HOXB3, HOXA3* | 24,07 | 1,000 |
| ***Cellular component*** |  |  |  |  |
| GO:0005634~nucleus | <0,001 | *ZNF831, HOXA11, SMYD3, GLDC, HOXB3, HOXA1, HOXB1, HOXB2, HOXA5, HOXB8, HOXA6, HOXB6, HOXA7, HOXA10, HOXA9, MLXIP, PSMD9* | 2,76 | 0,004 |
| GO:0005667~transcription factor complex | 0,008 | *HOXA11, HOXA10, HOXA9, HOXB13* | 9,45 | 0,281 |
| GO:0005721~pericentric heterochromatin | 0,033 | *KDM4C, CBX1* | 57,88 | 0,749 |
| ***Molecular function*** |  |  |  |  |
| GO:0043565~sequence-specific DNA binding | <0,001 | *HOXA1, ZNF831, HOXB1, HOXA3, HOXB2, EVX1, HOXB7, HOXA6, HOXA11, HOXB6, HOXA9, HOXB13* | 17,19 | <0,001 |
| GO:0003700~transcription factor activity, sequence-specific DNA binding | <0,001 | *ZNF831, HOXB2, HOXB7, HOXB8, HOXA6, HOXB6, MLXIP* | 6,97 | 0,011 |
| GO:0000978~RNA polymerase II core promoter proximal region sequence-specific DNA binding | 0,029 | *HOXA2, HOXA5, HOXA7, HOXA10* | 5,71 | 0,630 |
| GO:0001077~transcriptional activator activity, RNA polymerase II core promoter proximal region sequence-specific binding | 0,086 | *HOXA5, HOXA7, HOXA10* | 5,91 | 0,949 |
